# Beyond the Platinum Era—Scalable Preparation and Electrochemical Activation of TaS_2_ Flakes

**DOI:** 10.1021/acsami.2c20261

**Published:** 2023-01-20

**Authors:** Vladislav Buravets, Frantisek Hosek, Ladislav Lapcak, Elena Miliutina, Petr Sajdl, Roman Elashnikov, Václav Švorčík, Oleksiy Lyutakov

**Affiliations:** † Department of Solid State Engineering, University of Chemistry and Technology, 166 28 Prague, Czech Republic; ‡ Central Laboratories, University of Chemistry and Technology, 166 28 Prague, Czech Republic; § Department of Power Engineering, University of Chemistry and Technology, Prague 166 28, Czech Republic

**Keywords:** transition-metal dichalcogenides, CS_2_, sulfurization, TaS_2_, flakes, hydrogen evolution reaction, electrochemical activation

## Abstract

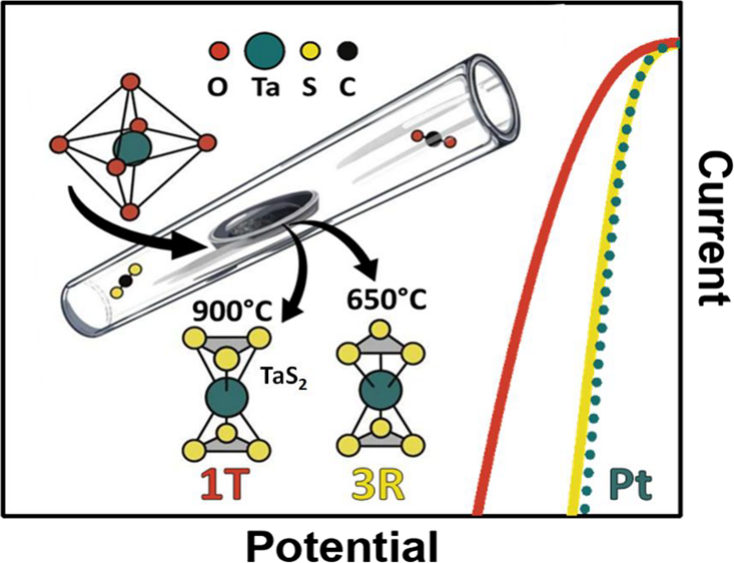

Among 2D materials, transition-metal dichalcogenides (TMDCs) of group 5 metals recently have attracted substantial interest due to their superior electrocatalytic activity toward hydrogen evolution reaction (HER). However, a straightforward and efficient synthesis of the TMDCs which can be easily scaled up is missing. Herein, we report an innovative, simple, and scalable method for tantalum disulfide (TaS_2_) synthesis, involving CS_2_ as a sulfurizing agent and Ta_2_O_5_ as a metal precursor. The structure of the created TaS_2_ flakes was analyzed by Raman, XRD, XPS, SEM, and HRTEM techniques. It was demonstrated that a tuning between 1T (metallic) and 3R (semiconductor) TaS_2_ phases can be accomplished by varying the reaction conditions. The created materials were tested for HER, and the electrocatalytic activity of both phases was significantly enhanced by electrochemical self-activation, up to that comparable with the Pt one. The final values of the Tafel slopes of activated TaS_2_ were found to be 35 and 43 mV/dec for 3R-TaS_2_ and 1T-TaS_2_, respectively, with the corresponding overpotentials of 63 and 109 mV required to reach a current density of 10 mA/cm^2^. We also investigated the mechanism of flake activation, which can be attributed to the changes in the flake morphology and surface chemistry. Our work provides a scalable and simple synthesis method to produce transition-metal sulfides which could replace the platinum catalyst in water splitting technology.

## Introduction

The global energy demands per year are between 18.5 and 20 Terawatt-years (TWyr), and about 84% of this energy is produced from gas, petroleum, and coal,^1,2^ while the estimated available amount of these sources is about 1400 TWyr.^1^ Utilization of alternative energy sources, like solar and wind energy, is debased by the absence of long-term energy storage.^3^ One of the most elegant solutions to surpass this drawback is energy storage in the form of chemical bonds, in particular through hydrogen production by, for example, water splitting. However, efficient water splitting depends on catalysts, commonly based on scarce elements,^4−6^ for example, Pt and Ir, which prevent the water splitting technology to be scaled up an industrial level. Thus, the search for efficient (photo)electrocatalysts, able to substitute Pt and Ir, is one of the most demanding areas of research.^7−10^

Theoretical screening of potential catalysts for hydrogen evolution reaction (HER), a cathodic reaction in the process of water splitting, suggests transition-metal dichalcogenides (TMDCs) as promising alternatives to state-of-the-art Pt-based catalysts.^11,12^ These assumptions have been confirmed experimentally, noting that a certain post-treatment is required to achieve a high electrocatalytic activity of TMDCs.^13−15^ For TMDC “activation”, various routes have been proposed, including mechanical, chemical, microwave, plasma-assisted ones, as well as a more simple and attractive electrochemical “self-activation” method under HER conditions.^12,16^ Despite the promising characteristics of TMDCs, the synthesis of these materials is not a trivial task.^17,18^ Although some of them can be synthesized by the solvo-/hydrothermal method, or more recently by CVD, most of them are still synthesized by temperature-driven reactions between elemental powders sealed under vacuum.^14,15,19^ Each of these techniques possesses some drawbacks such as low scalability, complicated setup, or poor crystallinity control. An alternative synthesis based on the sulfurization of transition-metal oxides by H_2_S, which can be potentially scaled up, was proposed earlier but was never adopted by the scientific society due to the complicated setup and high temperatures.^20^

Herein, we propose an easy method for the synthesis of TMDCs, demonstrated on the example of tantalum disulfide (TaS_2_). With carbon disulfide (CS_2_) as a strong sulfurizing agent, even oxides as a source of metal can be used in our method. Such an approach allows the significant reduction of the required temperature and simplification of the experimental equipment and makes the method potentially scalable.^21,22^ Under certain conditions, both 3R-TaS_2_ and 1T-TaS_2_ phases can be prepared. To test the theoretically estimated catalytic activity, the electrochemical response of the obtained phases of pristine and previously (electrochemically) activated TaS_2_ toward HER was also analyzed.

## Results and Discussion

**Figure 1:**
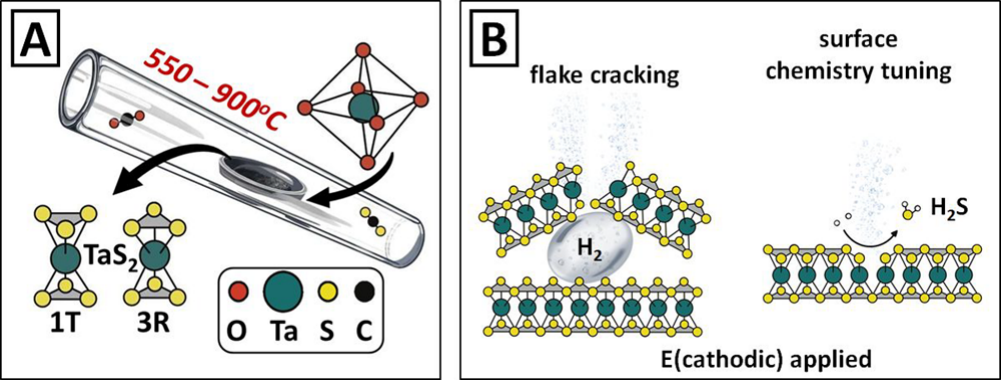
(A) Schematic representation of TaS_2_ synthesis by the sulfurization of Ta_2_O_5_ with the utilization of CS_2_ vapor flow at elevated temperatures—the creation of different TaS_2_ phases as a function of temperature is expected; (B) electrochemical TaS_2_ self-activation—application of negative potential results in hydrogen bubble appearance due to water splitting and bubble-induced flake cracking or S vacancy creation.

Our experimental approach is schematically shown in Figure 1A. Tantalum pentoxide was loaded into the reactor and heated in Ar atmosphere saturated with CS_2_ vapor (under a continuous flow), which acts as a sulfurizing and reducing agent (the chemical reaction equation is given in Scheme 1 in the Supporting Information). According to our assumption, the reaction of Ta_2_O_5_ and CS_2_ leads to the creation of TaS_2_ and formation of CO_2_ and S_2_ as byproducts that are removed from the reactor by the carrier gas.^23^ The first observed oxide changes were registered when the temperature was increased to 600 °C as the color transition from white to gray (550 °C temperature left the oxide untouched). The increase of the temperature to 650 °C changed the sample color into entirely black after 3 h of treatment. To investigate the whole phase temperature dependence, the temperature was further increased up to 900 °C (by 50 °C steps), and the composition, crystallinity, and morphology of the obtained materials were analyzed. After structure evaluation, the TaS_2_ samples with the best phase purity were self-activated electrochemically in the potentiostatic mode during the process of hydrogen evolution and subsequently tested as a catalyst for HER (Figure 1B). At this stage, flake cracking and vacancy formation were expected, both leading to a HER efficiency increase.

**Figure 2:**
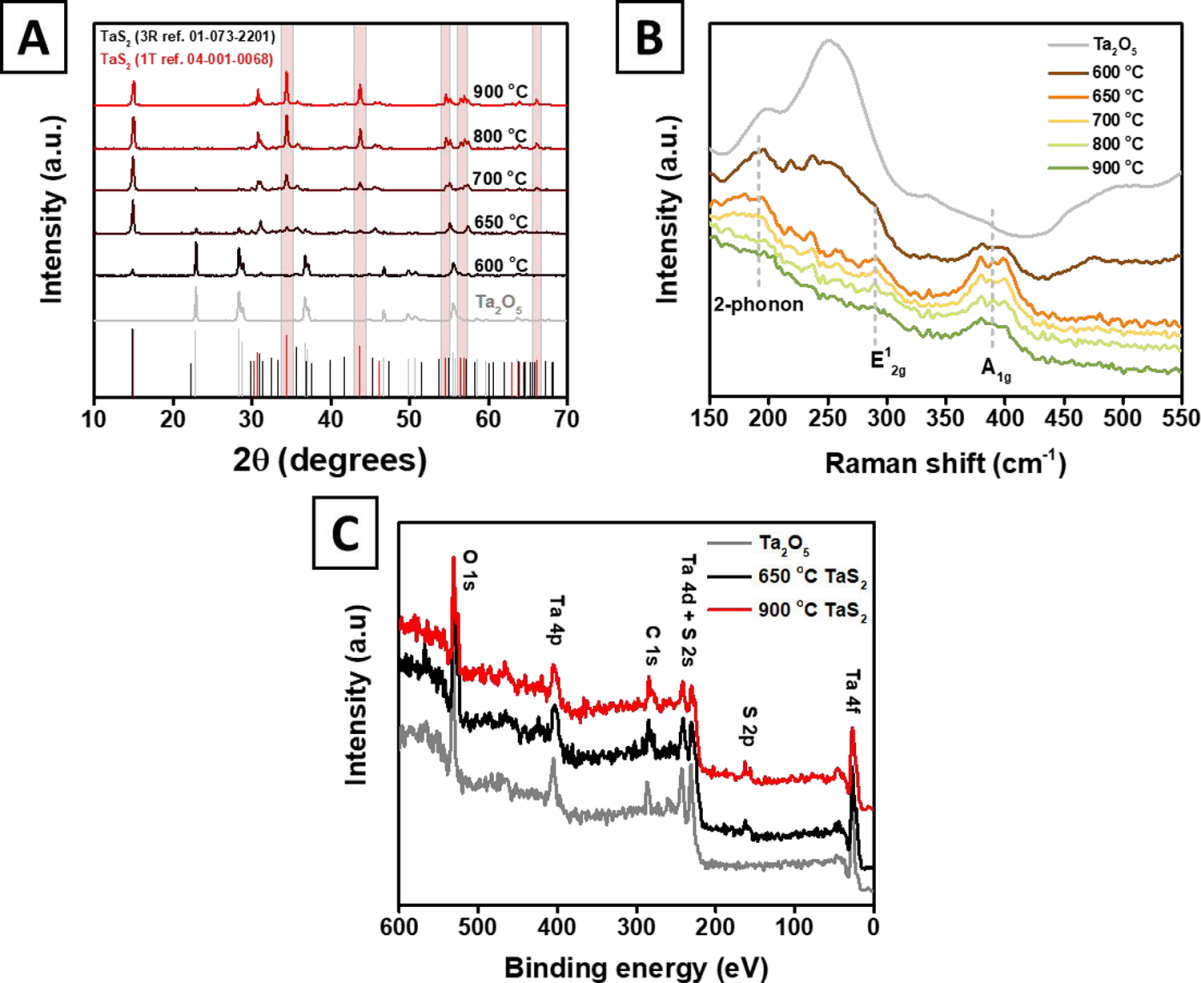
(A) XRD spectra of Ta_2_O_5_ and TaS_2_ flakes obtained after sulfurization at different temperatures for 3 h (the corresponding reference patterns are Ta_2_O_5_ ref. 01-089-2843; 3R-TaS_2_ ref. 01-073-2201; and 1T-TaS_2_ ref. 04-001-0068). (B) Raman spectra of Ta_2_O_5_ powder and TaS_2_ flakes synthesized at different temperatures (3 h in all cases), with the utilization of CS_2_ as a sulfurization agent. (C) XPS survey spectra of Ta_2_O_5_ and the two phases of TaS_2_ synthesized at 650 °C (3R phase) and 900 °C (1T phase).

The crystalline structure of the formed TaS_2_ was investigated as a function of reaction temperature and time by means of XRD and Raman (Figure 2) techniques. The XRD patterns of the as-synthesized materials in the temperature range between 550 and 900 °C along with the initial oxide are presented in Figures 2A and S1. After the synthesis at 600 °C for 3 h, characteristic peaks corresponding to the trace amounts of TaS_2_ become visible (3R phase, ref. 01-077-3359). However, the increase of the reaction temperature up to 650 °C accelerates the Ta_2_O_5_ conversion, and an almost complete absence of Ta_2_O_5_ and formation of 3R TaS_2_ phase are observed. In turn, at 700 °C and higher temperatures, the characteristic peaks of the 1T TaS_2_ phase (ref. 01-089-2843), highlighted in red, appear and become more and more prominent, suggesting that two mixed phases are formed at 700, 750, 800, and 850 °C. As the synthesis temperature is elevated to 900 °C, only small amounts of the 3R phase are present, so that 1T TaS_2_ comprises a major part of the resulting material (the relative phase ratio, obtained from XRD data, is presented in Figure S2). Changes in the peak positions between the 3R and 1T phases were observed and are shown in Figure S1. Similar peak positions were reported in the literature for the 3R^24,25^ and 1T^16,26,27^ phases. Raman spectroscopy also confirms that the sulfurization of Ta_2_O_5_ takes place at temperatures above 600 °C (Figure 2B), thus supporting the results of X-ray diffraction analysis. The resulting spectra show the suppression of the broad Raman band of Ta_2_O_5_ (250 cm^–1^) and the appearance of characteristic peaks at 390 and 290 cm^–1^, corresponding to the A_1g_ and E^1^_2g_ vibrational modes reported for different TaS_2_ phases.^12,28−32^ A broad peak at 180 cm^–1^ is commonly attributed to two phonon processes, which was observed for the trigonal crystal systems (H and R phases) of TaS_2_^24,30,31^ but was not generally observed for the hexagonal crystal system (T phase).^16,26,33−35^ A symmetry change for 1T-TaS_2_ was also reported earlier in the investigation of TMDCs by Raman spectroscopy.^36^ Thus, the absence of this peak from TaS_2_ synthesized at temperatures above 700 °C supports the XRD results that the 1T phase is preferentially formed at higher temperatures.

The synthesis at 600 °C for 3 h results in a spectral pattern, with the characteristic peaks of both tantalum sulfide and tantalum oxide. Further increasing the treatment time at 600 °C leads to an almost entire conversion to 3R-TaS_2_ after 16 h treatment, which was confirmed by XRD and Raman measurements (Figure S3). To confirm the chemical composition of the prepared samples, we have measured the X-ray photoelectron spectra of the samples synthesized at 650 and 900 °C and of tantalum pentoxide as well (Figure 2C). The survey spectra clearly show the appearance of the peaks at ∼163 eV (the position of S(2p) doublet in TaS_2_^16,25,29,31^), confirming the tantalum sulfurization. As the melting points of the Ta precursor as well as the formed TaS_2_ are much higher than the temperature in the reactor (1872 and 3000 °C, correspondingly), we believe that sulfurization proceeds through a solid-phase reaction. It is assumed that the reaction occurs on the oxide–vapor interphase and is followed by the gradual diffusion/migration of sulfur atoms inside the Ta_2_O_5_ powder and oxygen atoms toward the surface of Ta_2_O_5_ grains. This process is accompanied by the crystalline symmetry changes and formation of TaS_2_, which also depend on the applied temperature. However, it should be noted that the solid-phase processes are commonly considered to depend on many parameters, such as the reducing agent and its reducing ability, temperature, heating mode, presence of additives, component migration and diffusion, and so forth.^37,38^ Thus, more precise estimation of the TaS_2_ growth mechanism requires a more serious study (which could be the subject of our future research, but at this stage, it exceeds the scope of this work). Based on the abovementioned results, we chose two synthesis temperatures, namely 650 and 900 °C, and 3 h treatment time (further referred as 3R-TaS_2_ and 1T-TaS_2_ materials) for subsequent material characterization and utilization.

**Figure 3:**
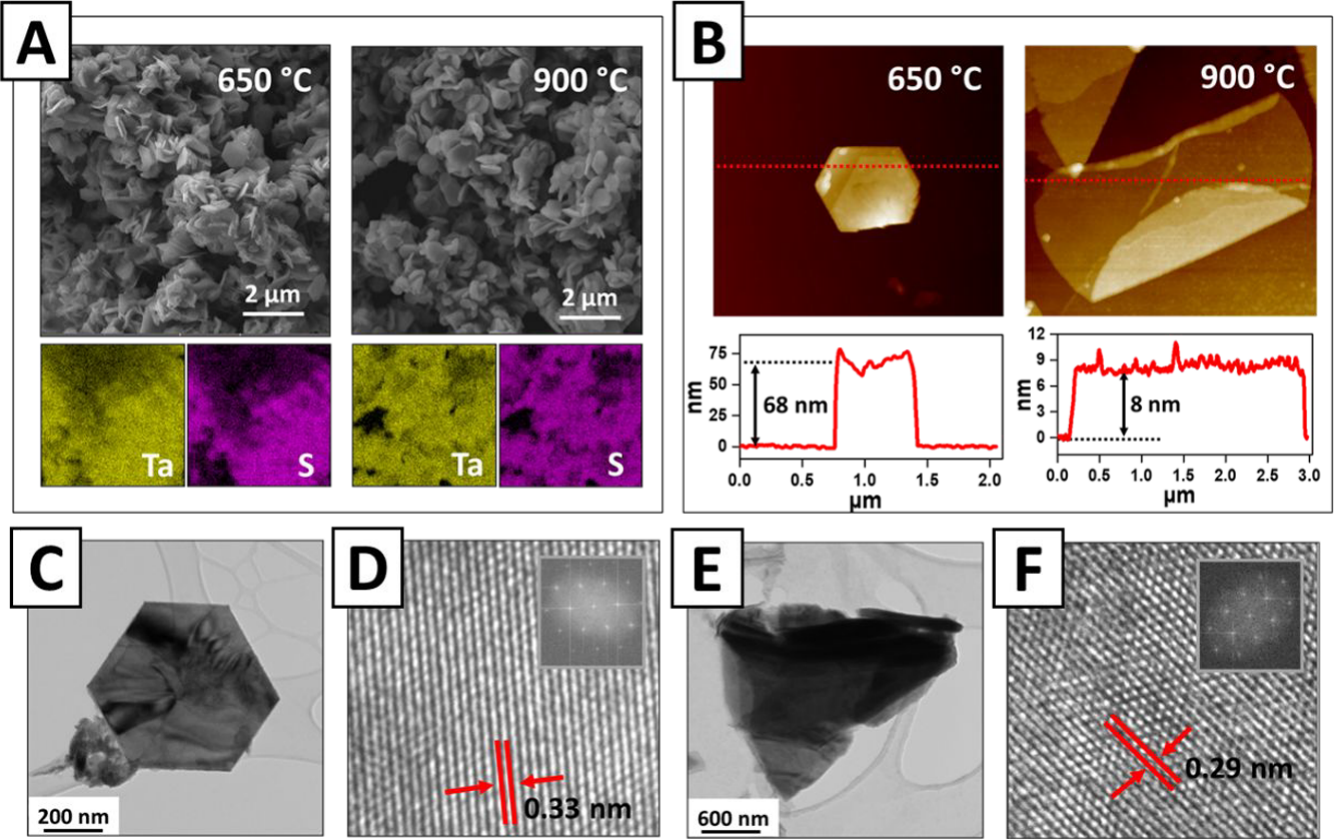
(A) SEM images (top) and EDX mapping (bottom) of 3R-TaS_2_ and 1T-TaS_2_ flakes synthesized at 650 and 900 °C; (B) AFM images (top) and the corresponding profiles (bottom) of 3R-TaS_2_ (obtained at 650 °C) and 1T-TaS_2_ (obtained at 900 °C) flakes after sonication and deposition on the silicon substrate; (C,D) TEM and HRTEM morphology and crystalline structure of a typical 3R-TaS_2_ flake; (E,F) TEM and HRTEM morphology and crystalline structure of a typical 1T-TaS_2_ flake (insets in D and F reflect the fast-Fourier transformation of the selected area).

Further morphological and structural analyses of the obtained TaS_2_ samples were performed by SEM, AFM, TEM, and HRTEM methods (Figure 3). The SEM images reveal the transition from the globular morphology of Ta_2_O_5_ to flake-like morphology, typical for both the phases of TaS_2_ (Figures 3A and S4). Moreover, the “layered” structure of the created material is also visible in the case of both TaS_2_ phases. The conversion of tantalum oxide into sulfide was also confirmed by EDX mapping (corresponding to the SEM image), indicating that flakes are composed of Ta and S, with an almost complete absence of oxygen-related signals (Figures 3A and S5). After the ultrasonication and re-deposition of the flakes (which can result in the delamination of both phases of TaS_2_) on the silicon surface, the AFM results show a hexagonal structure of the obtained 3R phase (Figure 3B). The created hexagonal 3R flakes are approximately 500 nm in diameter and 54.5 ± 12.6 nm thick. The observed flake(s) hexagonal geometry is in good agreement with the symmetry of the crystal lattice of the 3R-TaS_2_ phase. In turn, for the 1T phase, an alternative flake morphology was often observed (Figure 3B). In this case, an apparent tendency of the formation of flakes with significantly larger lateral dimensions is evident. 1T-TaS_2_ flakes are approximately 3 μm in length and 9.3 ± 1.8 nm thick (the formed flakes tended to curl around the edges (Figure 3B); thus, several additional AFM measurements were performed to evaluate the flake thickness with a higher accuracy (Figure S6)), the dimensions of which are closer to that of typical 2D materials. In turn, the TEM and HRTEM images (Figure 3C–F) confirm the typical flake morphology. From the images, the d-spacings were determined to be 0.33 nm for 3R-TaS_2_ and 0.29 nm for 1T-TaS_2_, both in agreement with the previously published results.^27,28,39^ Finally, the results of electron diffraction confirm the good crystallinity of both 3R-TaS_2_ and 1T-TaS_2_ flakes (Figure S7).

It is also worth noting that the proposed approach is favored by simplicity and potential scalability. The commonly used methods of TaS_2_ or other TMDC preparation include the CVD-based preparation or synthesis in a closed ampoule (Table S1). In these cases, the commonly used methods are somewhat limited by sophisticated equipment and the amount of material that can be obtained. In our case, the synthesis was carried out in a flow reactor, which imposes no restrictions on either the initial oxide loading or the amount of TaS_2_ obtained. For a more convincing demonstration, we performed an additional TaS_2_ synthesis (preparation of a more catalytically interesting 3R phase was chosen) with a 10-fold increase in the initial Ta_2_O_5_ loading (Figure S8). Even in this case, a 100% conversion of tantalum pentoxide to tantalum sulfide was observed, which confirms our assumption about the scalability of the proposed approach.

**Figure 4:**
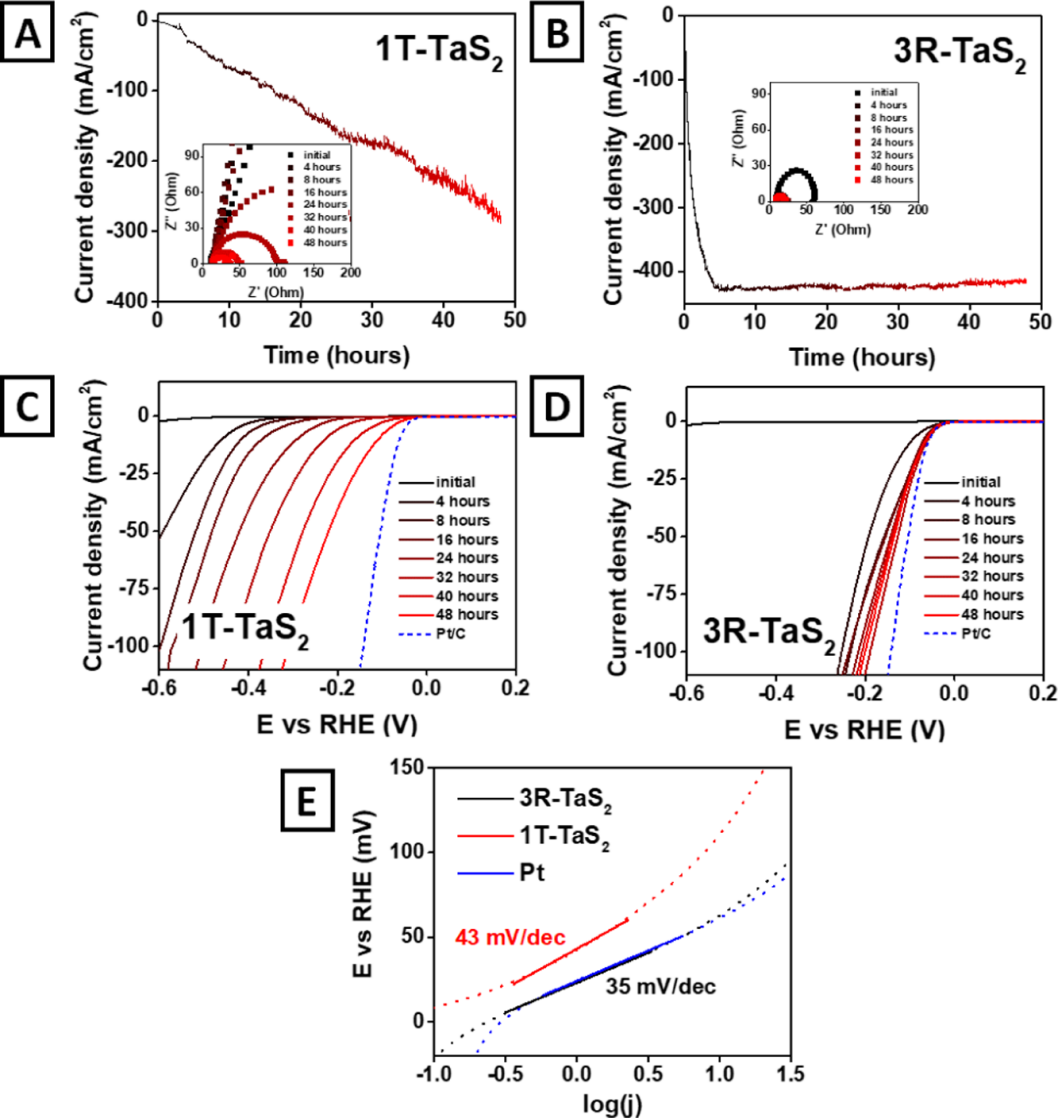
Electrochemical (potentiostatic) self-activation at −0.481 V vs RHE in 0.5 M H_2_SO_4_ of (A) 1T-TaS_2_ phase prepared at 900 °C and (B) 3R-TaS_2_ flakes, prepared at 650 °C; the insets show the corresponding Nyquist plots. (C,D) LSV curves, measured after different times of 3R-TaS_2_ (C) and 1T-TaS_2_ (D) electrochemical self-activation in the HER regime, (E) estimated from the LSV curve values of Tafel slopes of activated 3R-TaS_2_ and 1T-TaS_2_ flakes.

In the next step, the catalytic activity toward HER for the synthesized 1T-TaS_2_ and 3R-TaS_2_ was investigated. The as-prepared 1T- and 3R-TaS_2_ show a low HER catalytic efficiency (Figure S9 represents the LSV curves of 1T and 3R phases in comparison with Pt, as a “gold” standard in HER catalysis). Thus, a subsequent electrochemical activation was performed to enhance the electrocatalytic activity.^40−42^ In particular, 1T-TaS_2_ and 3R-TaS_2_ flakes were self-activated in the potentiostatic mode at −0.481 V vs RHE in 0.5 M H_2_SO_4_ (Figure 4,BA). As can be seen from the chronoamperometric curves, the initial current density at a given potential was negligibly low for both phases. However, the gradual catalyst treatment leads to the significant (by several orders of magnitude) increase of the current density. For 1T-TaS_2_, the time dependence of the current was linear, while for 3R-TaS_2_, it was rather exponential it reaches a plateau of ∼420 mA/cm^2^ after approximately 4–8 h of activation. By contrast, 1T-TaS_2_ does not achieve a current density plateau even after 48 h of activation (reaching a current density of ∼260 mA/cm^2^). LSV and EIS measurements, performed every 4 h of activation, also well reflect the increase of the catalytic activity of TaS_2_ (Figure 4C,D). Activation changes the LSV profile, and a shift in the anodic direction is observed for both phases. For 3R-TaS_2_, a significant change was observed already after 4–8 h of activation, and the measured LSV curves approach the Pt standard (Figure 4D). In turn, LSVs recorded on 1T-TaS_2_ show a more continuous change during the activation process. From the obtained curves, the overpotential required for the current density of 10 mA/cm^2^ was determined as 109 mV for 1T-TaS_2_ and 63 mV for 3R-TaS_2_. It follows from the EIS measurements (insets in Figure 4A,B) that because of the activation, the diameter of the semicircle diminishes for both phases, which indicates a decrease in the resistance for HER and, consequently, facilitates an electron transfer. The change dynamics also indicates that the charge-transfer facilitation for the 1T phase occurs throughout the entire activation period, while for the 3R phase, it reaches a constant value in the interval of 4–8 h of activation. Based on the LSV measurements, Tafel slopes were calculated for the pristine and activated TaS_2_ phases (Figures 4C,D and S9). The slopes of the as-synthesized 1T-TaS_2_ and 3R-TaS_2_ were found to be 160 and 135 mV/dec, respectively, which are very far from that of the “ideal” catalyst (Figure S10). However, after the activation, the Tafel slopes for both phases (1T and 3R) were reduced significantly to 43 and 35 mV/dec, respectively (Figure 4E). We also performed the cyclic stability tests for both the 3R-TaS_2_ and 1T-TaS_2_ phases after their activation by performing cyclic voltammetry for 1000 cycles (Figure S11). We have observed some increase in current after this procedure for the 1T-TaS_2_ phase, which can be expected due to the additional activation of these flakes. In the case of 3R-TaS_2_, a slight decrease of current density was observed, which can be attributed to the peeling of the material from the electrode surface.

**Figure 5:**
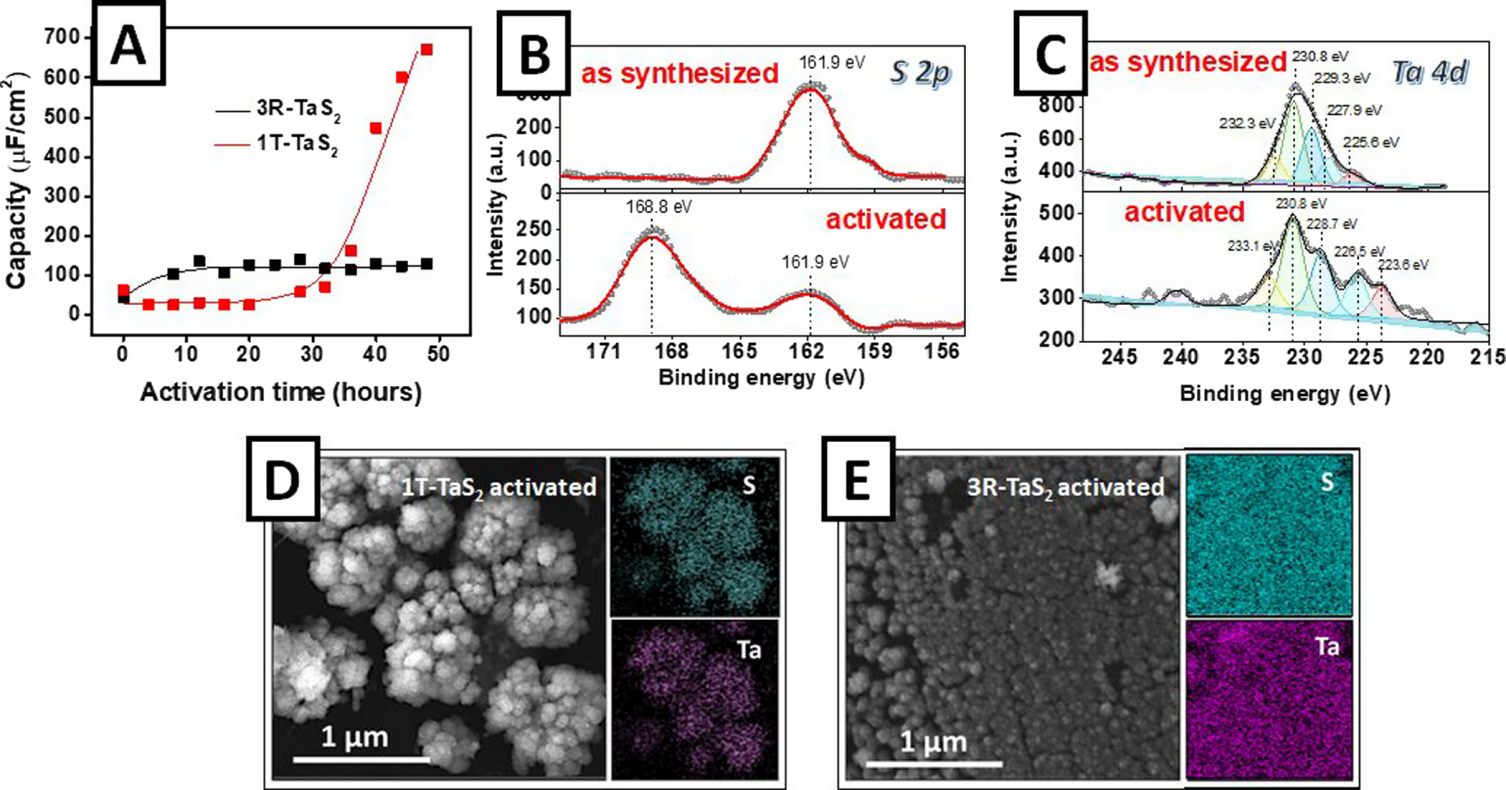
(A) Estimation of ECSA: capacitance change in the process of self-activation of 3R-TaS_2_ and 1T-TaS_2_ flakes in the HER regime; (B,C) high-resolution S (2p) and Ta (4d) XPS spectra before and after the electrochemical self-activation of 3R-TaS_2_ flakes; (D,E) SEM images of activated 1T-TaS_2_ and 3R-TaS_2_ flakes and the corresponding EDX mapping of Ta and S.

The observed HER efficiency enhancement and the corresponding decrease of the Tafel slope values can be explained by the combination of the following phenomena: (i) cracking of the flakes, which increases the number of catalytically active edges,^12^ (ii) flake exfoliation, which leads to a lesser number of layers for the charge to pass through,^13^ and (iii) formation of catalytically active vacancies on the flakes’ basal plane.^39^ To investigate the activation mechanism, we have analyzed the double-layer capacitance, as it is related to the electrochemically active surface area (ECSA). Cyclic voltammograms were recorded (Figures S12–S14 and the related discussion in the Supporting Information), and the evolution of the surface capacity value during the TaS_2_ activation is presented in Figure 5A. After 12 h of activation of 3R-TaS_2_, its capacitance increases 3.2 times from the initial 43 μF/cm^2^ to the maximum 138 μF/cm^2^. However, such a change of the surface area itself could not explain the increase of the current density by 2 orders of magnitude from ∼2 to ∼420 mA/cm^2^ after 8 h of activation. In contrast, the double-layer capacitance of 1T-TaS_2_ does not change significantly during the first ∼20 h of activation, while a more than 25-fold increase in capacitance was observed during subsequent activation. Such a behavior does not correlate with the trends of electrocatalytic performance enhancement (Figure 5A vs Figure 4A). Therefore, in both the 3R-TaS_2_ and 1T-TaS_2_ phases, the significant enhancement of the catalytic activity cannot be explained by the increase of ECSA.

To further investigate the changes occurring during the activation, XPS and SEM measurements were performed. The survey XPS spectra (Figure S15) reveal the apparent decrease of TaS_2_ surface concentration, which reveals the catalyst removal from the surface due to flake delamination and potentially facilitates electron transport through the flake(s) layer. The high-resolution XPS spectra of sulfur and tantalum (measured on the “more interesting” 3R-TaS_2_ phase) are presented in Figure 5B,C (Figure S16 provides the same information for the 1T-TaS_2_.phase). In Figure 5B, an additional sulfur peak at higher binding energies corresponding to S–O bonds^16,31,43,44^ is visible. The peak is more pronounced for the 3R phase than for the 1T one (Figure 5B vs Figure S16A,C), suggesting that higher amounts of S–O bonds were formed on 3R-TaS_2_. The primary catalytic role of the formed S–O bond is unknown, but we estimate that its appearance is related to edge formation. When the number of edges increases, more unsaturated S and Ta bonds are left, which are prone for further oxidation after the potential is released. This assumption is in accord with the shift of Ta 4d peaks toward higher binding energies and thus higher oxidation states (see Figures 5C and S16B,D). Finally, the SEM images (Figures 5D,E and S17) measured before and after the activation of both 3R-TaS_2_ and 1T-TaS_2_ clearly show the reduction of the nanostructure size during activation. The corresponding mapping of Ta and S surface distribution confirms that the resultant nanoparticles are in fact fractures of the initial TaS_2_. EDX mapping also indicates some increase of surface oxygen concentration after flake utilization in HER (Figure S18). It can be seen that the 3R phase was crashed into particles of smaller size, the finding which correlates well with higher catalytic activity and the discussed XPS spectra. Thus, considering the obtained results, we estimate that cracking of the TaS_2_ flakes plays a significant role in the activation process.

## Conclusion

In summary, the synthesis method of TaS_2_ flakes using metal oxide as the metal precursor and CS_2_ as the sulfurizing agent was developed. The advantages of this method in comparison to other ones is in its simplicity, scalability, phase control, and comparably low treatment temperatures. Adjusting the synthesis temperature allows the controllable formation of 3R-TaS_2_ phase (≤650 °C) and 1T-TaS_2_ (900 °C). The electrocatalytic activity of the synthesized 3R-TaS_2_ and 1T-TaS_2_ was investigated toward HER. It was shown that the catalytic performance of both phases can be significantly increased by their electrochemical self-activation at the potentiostatic mode. 3R-TaS_2_ outperforms 1T-TaS_2_ in the catalytic activity toward HER; moreover, it shows the catalytic activity comparable with Pt. We also investigate the flake activation mechanism and suggested that it correlates with flake cracking and delamination, which results in the increase in the electrochemically active sites, namely edges, and facilitates electron transfer. We did not observe the appearance of sulfur vacancies in the TaS_2_ structure, which could be obscured by the significant morphological changes. Finally, it should be noted that our synthesis method is simple and potentially scalable, representing an alternative and attractive route for the creation of TMDC catalysts for rare metal-free water splitting.

## Experimental Section

### Materials and Sample Preparation

The detailed description of the materials used and experimental procedures are given in the Supporting Information. Briefly, TaS_2_ was synthesized in a quartz tube (previously filled with inert gas) from Ta_2_O_5_ powder under elevated temperatures and using CS_2_ vapor brought to the reacting atmosphere by Ar. The sketch of a home-made experimental setup is shown in Figure S19 (with enclosed detailed description). Note that due to the toxicity of CS_2_, careful handling is required, and all manipulations must be performed in the fume hood. Residual gases were trapped by bubbling through the NaOH solution. The synthesis was performed in a range of temperatures (550–900 °C), for 3 h, unless otherwise specified, with 10 °C/min heating rate. As-synthesized TaS_2_ was deposited on a glassy carbon surface and activated electrochemically in potentiostatic mode at a constant potential of −0.481 V vs RHE within 48 h. Electrochemical impedance spectroscopy (EIS), linear sweep voltammetry (LSV), and cyclic voltammetry (CV) were recorded every 4 h of activation.

### Characterization

A detailed description of the characterization techniques used is given in the Supporting Information. All the electrochemical experiments and measurements were performed in a three-electrode system controlled by PalmSens4, using Ag/AgCl as the reference electrode, Pt (or carbon rod) as the counter electrode, and glassy carbon (3 mm in diameter) as the substrate for the working electrode, in a 0.5 M H_2_SO_4_ electrolyte purged with N_2_. The LSV scan rate was set to 10 mV/s, while CV measurements were recorded for the estimation of the double-layer capacitance *C*_dl_ in the range 20–110 mV/s with a step of 10 mV. EIS was measured in the range between 0.1 Hz and 100 kHz, at an applied potential of −0.131 V vs RHE and alternating it by the value of 10 mV. Potentiodynamic probing (cyclic stability test) was performed by running cyclic voltammetry for 1000 cycles at a scan rate of 50 mV/s in 0.5 M H_2_SO_4_ in the potential range from 0.22 to −0.58 V vs RHE.

## Supporting Information Available

Chemical reaction equation of Ta_2_O_5_ sulfurization;_._materials and reagents, synthesis setup, characterization methods, sample preparation, additional XRD and XPS patterns, SEM, AFM, EDX, and TEM images; materials and phase compositions of Ta_2_O_5_ sulfurization product(s); LSV plots and Tafel slopes of as-synthesized TaS_2_; CV plots for ESCA estimation; and cyclic stability test performed in CVA regime for previously activated 1T-TaS2 and 3R-TaS2 phases (PDF)
