# Supplementary material for: Beyond the Platinum Era—Scalable Preparation and Electrochemical Activation of TaS2 Flakes
Source: ACS Appl Mater Interfaces. 2023 Jan 20;15(4):5679–86. doi: 10.1021/acsami.2c20261 (PMC10016745; doi:10.1021/acsami.2c20261)
Supplement: Supplementary file 1 — am2c20261_si_001.pdf [file am2c20261_si_001.pdf]

Supporting information for:

"Beyond the platinum era – scalable preparation and electrochemical activation of TaS_2 flakes" by

Vladislav Buravets[a], Frantisek Hosek[a], Ladislav Lapcak[b], Elena Miliutina[a], Petr Sajdl[c], Roman Elashnikov[a], Václav Švorčík[a], Oleksiy Lyutakov[a,*]

[a]*Department of Solid State Engineering, University of Chemistry and Technology, 166 28 Prague, Czech Republic*

[b]*Central Laboratories, University of Chemistry and Technology, 166 28 Prague, Czech Republic*

[c]*Department of Power Engineering, University of Chemistry and Technology, Prague 16628, Czech Republic*

Corresponding author: lyutakoo@vscht.cz

Experimental part

Materials and reagents

Tantalum pentoxide (Ta_2O_5, 99.99 %, < 20 μm), Carbon Disulfide (CS_2, 99.9 %) were purchased from Sigma-Aldrich. Ar (6.0 grade) was provided by SIAD. Isopropyl alcohol (99.96 %) was supplied by Lach-Ner. NaOH. H_2SO_4. A 5 % solution of Nafion 117 was purchased from Sigma-Aldrich.

Sample preparation

To perform electrochemical measurements glassy carbon (BASi) with diameter of 3 mm was used as a substrate. To deposit catalyst on the surface, suspension was prepared by adding 10 mg of as synthesized TaS_2 into 5 ml of isopropanol: water (4:1) mixture with addition of 20 μL of Nafion and dispersed for 10 min in ultrasonic bath. To form a catalytically active layer 10 μL of the obtained suspension was dropcasted on the surface of the glassy carbon and dried in air for 30 min.

Characterization methods

XRD analysis was carried out with the use of PanAnalytical X´pert Pro, with Cu Kα 1.54 Å wavelength excitation. The XRD data were analysed using HighScore Plus software to reveal the relative phase ratio and the composition of sulfurization product(s). Atomic force microscopy was performed by Bruker Icon Dimension AFM. Flakes thickness estimation was performed using 10 (3R-TaS_2) and 30 (1T-TaS_2) AFM scans and it is given as an averaged value with corresponded statistical deviation. Scanning electron microscopy coupled with energy dispersive X-ray spectroscopy (SEM/EDX) analysis was performed using LYRA3 GMU by Tescan. Raman spectra

were collected on Thermo Scientific DXR Raman Microscope with a 532 nm laser excitation. High resolution transmission electron microscopy (HR-TEM) and selected area electron diffraction (SAED) analysis were performed on Jeol JEM-2200 FS. The XPS survey and high-resolution spectra were obtained using an Omicron Nanotechnology ESCAProbeP spectrometer. XPS analysis was performed under ultra-high vacuum using a monochromatic Al K Alpha X-ray source. The acquired XPS spectra were evaluated by CasaXPS software. The acquired XPS spectra were evaluated by CasaXPS software, spectra were corrected to compensate surface charging.

$$2Ta_2O_5 + 5CS_2 \rightarrow 4TaS_2 + 5CO_2\uparrow + S_2\uparrow$$

Scheme 1 Chemical reaction equation of Ta_2O_5 sulfurizing by CS_2.

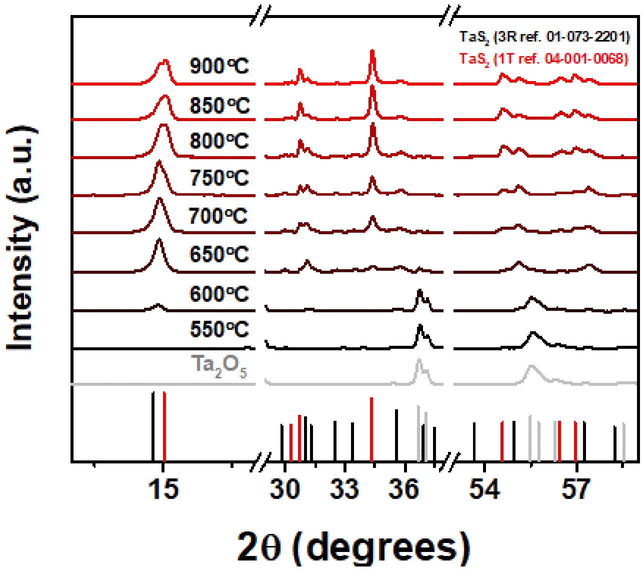

Figure S1 Selected area XRD patterns of Ta_2O_5 and as-synthesized TaS_2 at different temperatures for 3 hours.

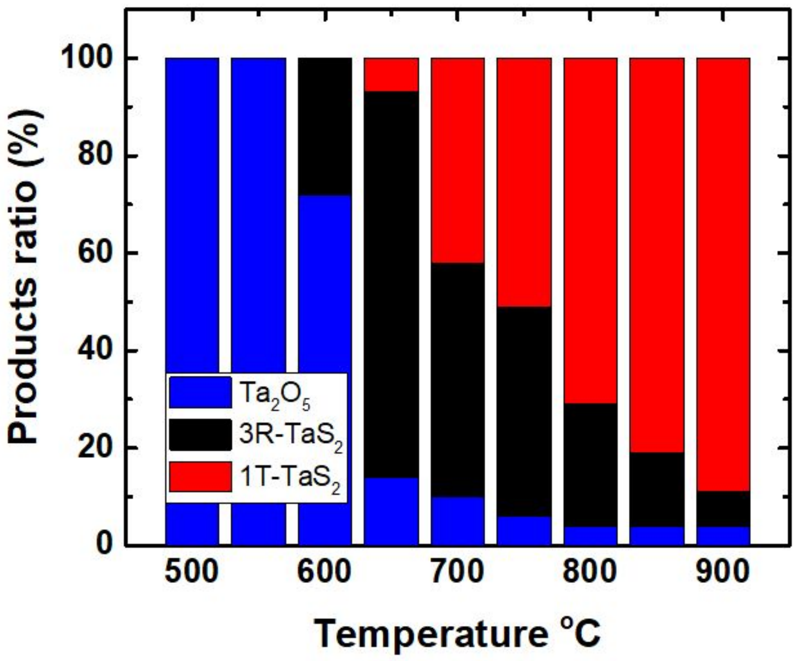

Figure S2 Materials and phase compositions of Ta2O5 sulfurization product(s) as a function of reaction temperature (duration of sulfurization – 3h. in all cases).

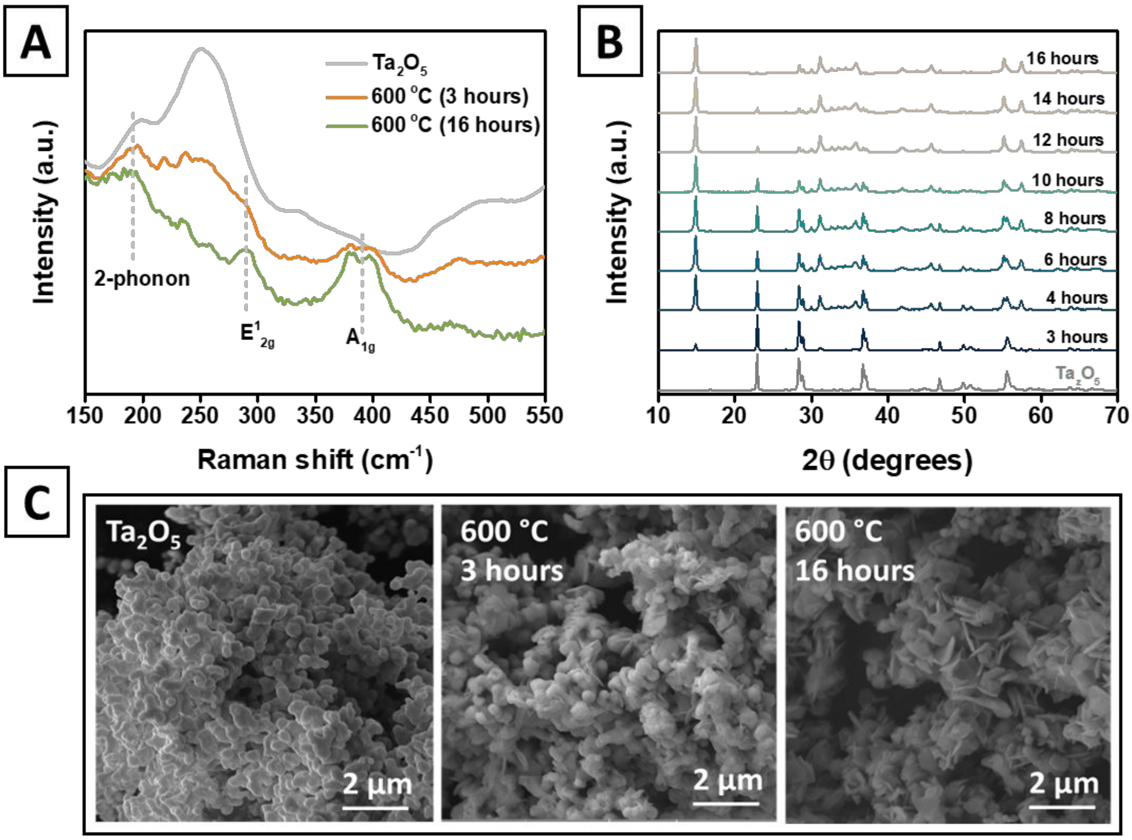

Figure S3 (A) – Raman spectra of pristine Ta_2O_5 and $TaS2_2$ synthesized at 600 °C during 3 and 16 hours; (B) - XRD spectra of Ta_2O_5 and obtained TaS_2 after sulfurization at 600 °C for different times; (C) – SEM images of pristine Ta_2O_5 and TaS_2 synthesized at 600 °C temperatures during 3 and 16 hours.

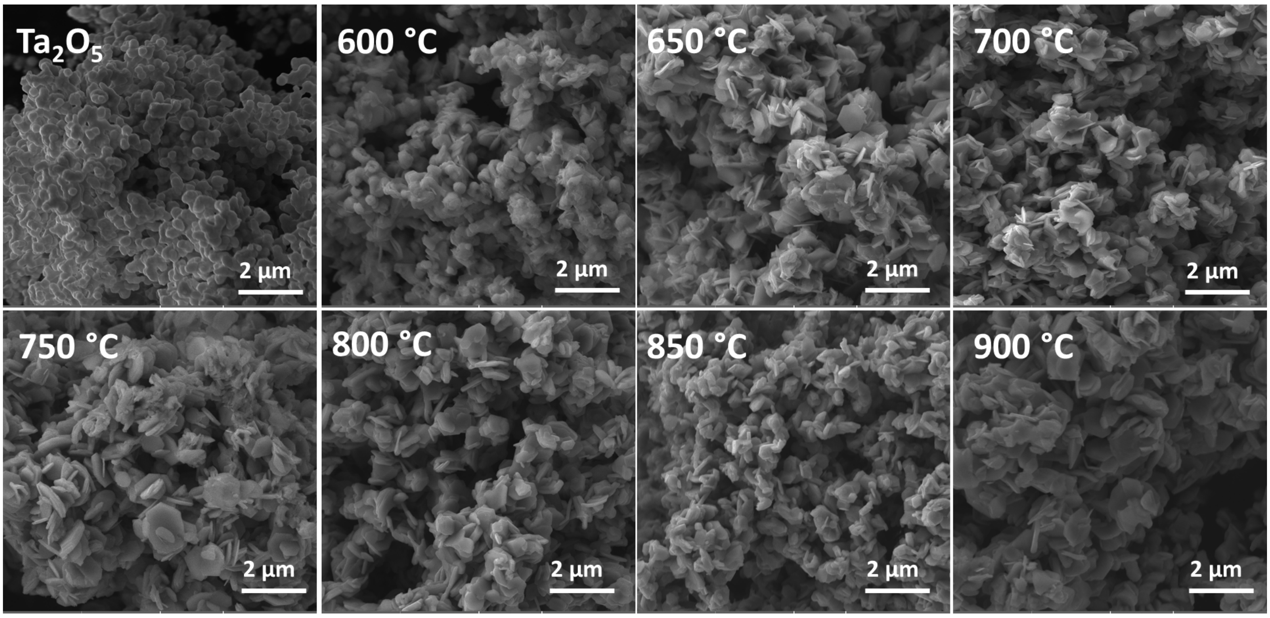

Figure S4 SEM images of Ta_2O_5 and as-synthesized TaS_2 at different temperatures set for 3 hours.

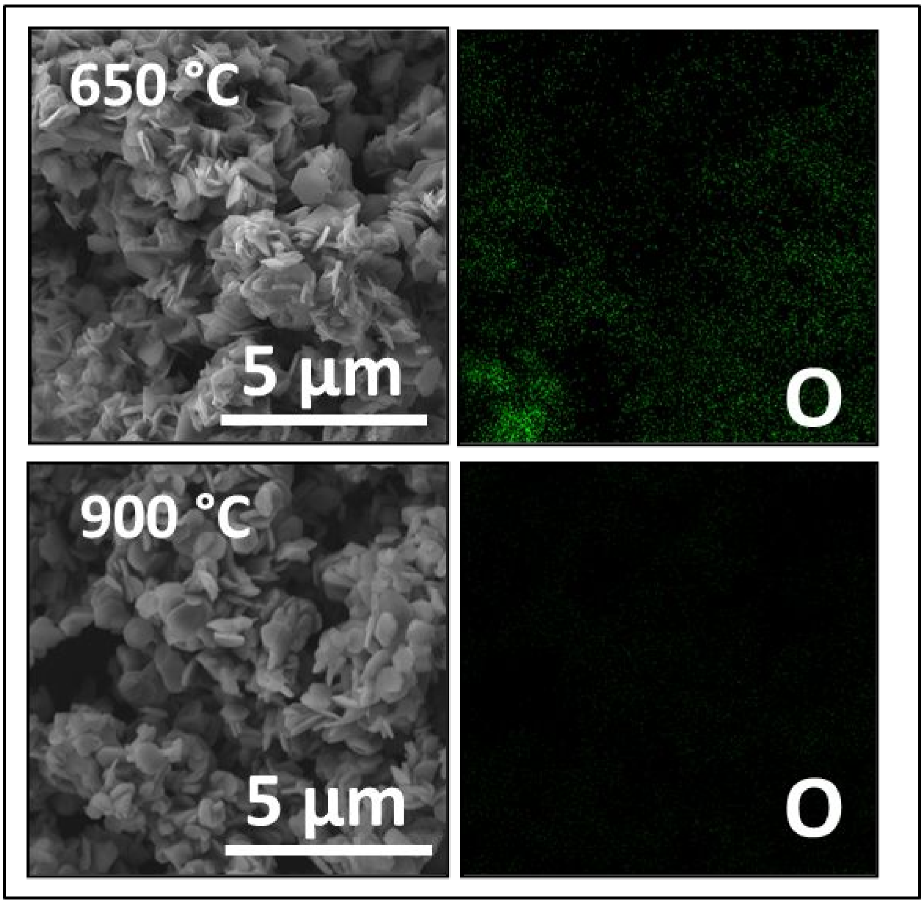

Figure S5 Additional EDX mapping (oxygen content) of 3R-TaS_2 and 1T-TaS_2 flakes synthesized at 650 and 900 °C.

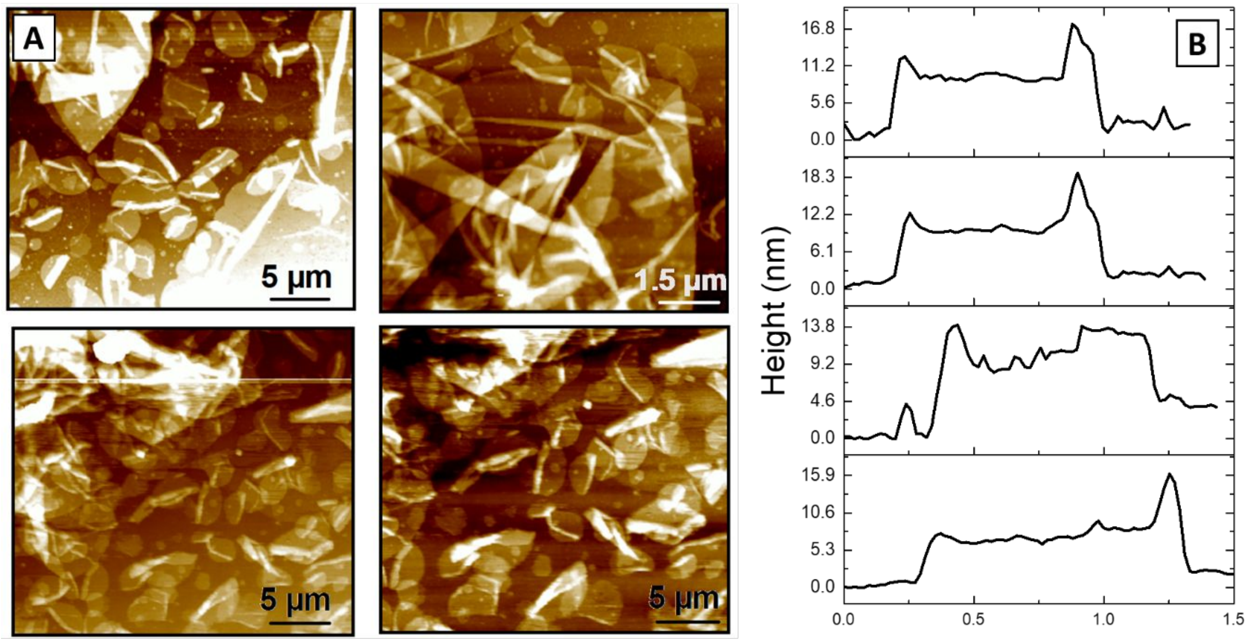

Figure S6 AFM images (A) and corresponding profiles (B) of 1T-TaS_2 (obtained at 900 ^oC) flakes after sonication and deposition on silicon substrate.

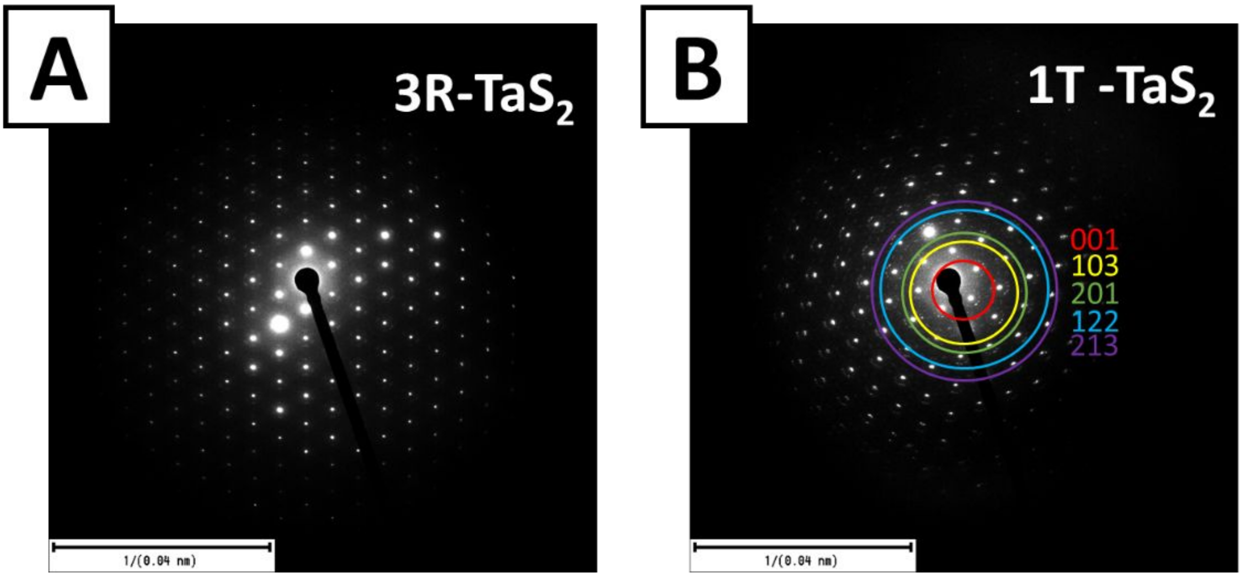

Figure S7 Selected area electron diffraction patterns of as synthesized 3R-TaS_2 (A) and 1T-TaS_2 (B).

Figure S7– related discussion. Resulted SAED patterns of as synthesized 3R-TaS_2 and 1T-TaS_2 show only one set of hexagonally arranged diffraction spots, which confirms high crystallinity of the obtained materials in accordance with the FFT images (main text).

Table S1 Comparison of proposed approach with previously published ones.

Synthesis method	Precursors	temperature	time	phase	reference
Evacuated quartz tube	elemental Ta and S	900 °C	several days	2H	S1
Two zone furnace, in Ar + H2	laser treated Ta-foil to form oxide layer, elemental sulfur	180 and 900 °C	3 hours	3R	S2
Evacuated quartz ampoule	elemental Ta and S (+iodine)	600, 800, 1000 °C for 1st step and 950 °C for 2nd step	150 hours (1st step) + 7 days (2nd step)	1T	S3
Evacuated quartz tube, two zone furnace	elemental Ta and S (+iodine)	850 °C (1st step) and 800-900 °C (2nd step)	4 days (1st step) + 2 weeks (2nd step)	1T	S4
Evacuated quartz ampoule	elemental Ta and S	450 °C (1st step), 600 °C (2nd step) 900 °C (3rd step)	12, 48 and 48 hours for 3 steps, respectively	6R	S5
Sealed vacuum quartz tubes	elemental Ta and S (+iodine)	gradient 730-770 °C	21 days	2H	S6
Evacuated quartz container	elemental Ta and S	450 °C (1st step), 600 °C (2nd step), 900 °C (3rd step), cooling (4th step)	12, 48, 48 and 24 hours for 4 steps, respectively	H	S7
Sealed vacuum quartz tubes	elemental Ta and S	900 °C	72 hours	1T	S8
Evacuated quartz tube	elemental Ta and S	1000 °C	4 days	2H	S9
Chemical vapor deposition in argon	TaCl5 and elemental S	750 °C	NaN	2H	S10
Two-zone chemical vapor deposition	TaCl5 and elemental S	230 and 750 °C	5-60 minutes	2H	S11
Chemical vapor deposition in argon/hydrogen	TaCl5 and elemental S	600 °C	5-7 minutes	1T	S12
Sealed vacuum quartz tubes	elemental Ta and S	900 °C	1 day	1T	S13
Two zone furnace, under vacuum	elemental Ta and S	827 °C	2 hours	3R	S14
Flow reactor (Ar+CS2)	Tantalum oxide	600-900 °C	3 hours	3R and 1T	this work

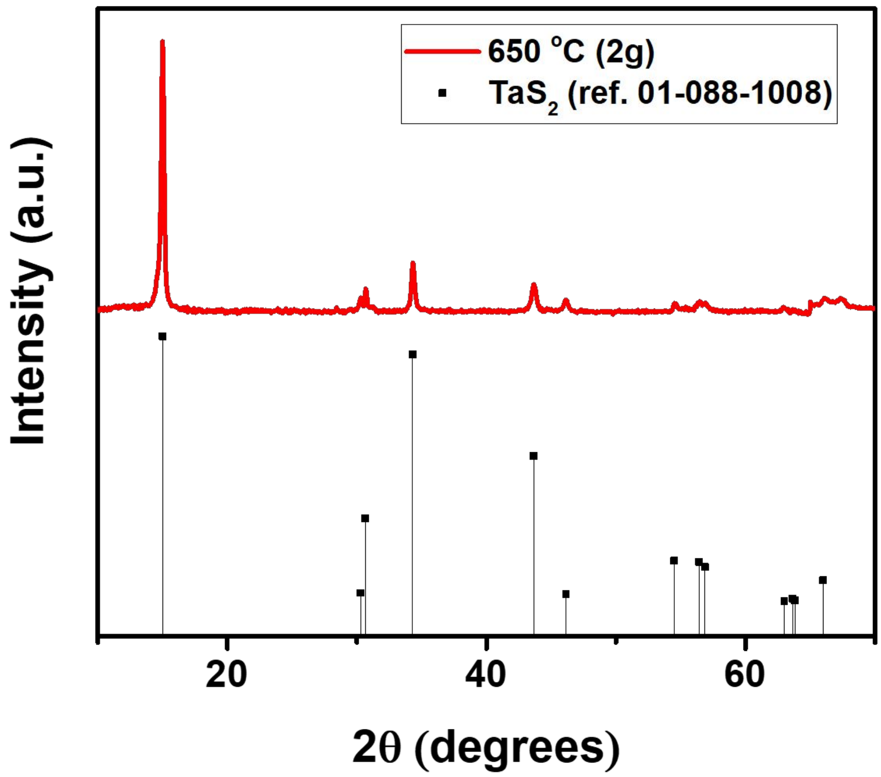

Figure S8 XRD spectra of TaS_2 flakes (3R phase, ref. 01-073-2201) obtained at 650 °C with utilization of 10x higher amount of initial Ta_2O_5 powder (compare to main results, described in manuscript) - complete absence of characteristic oxide peak indicates the closed to 100% conversion.

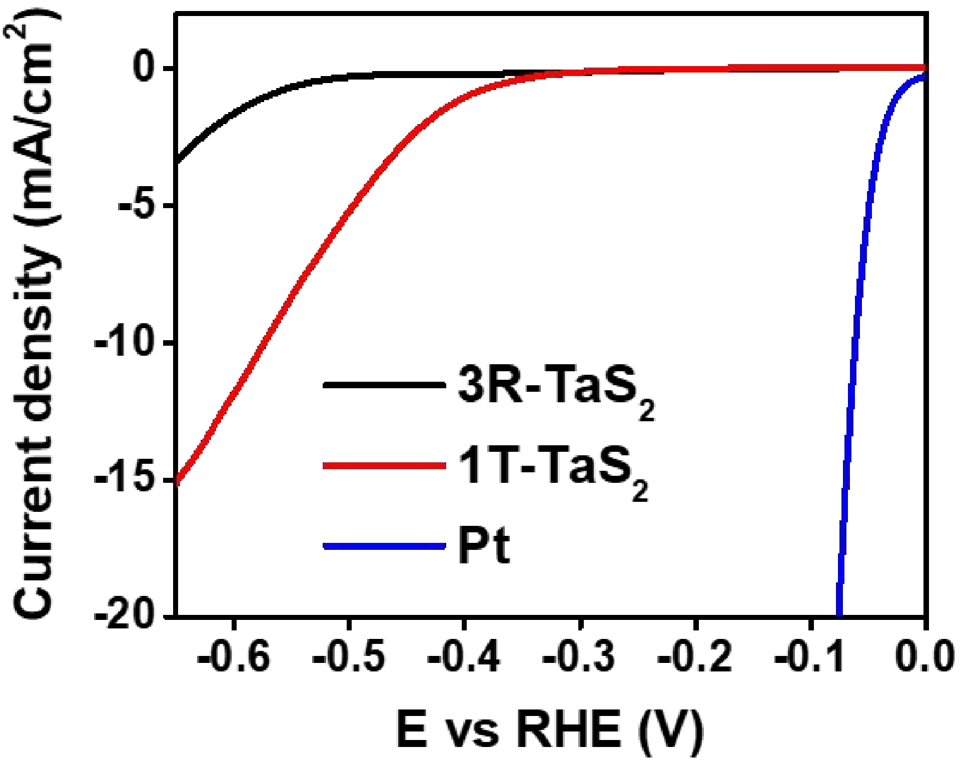

Figure S9 LSV curves of non-activated 1T and 3R phases in comparison with Pt.

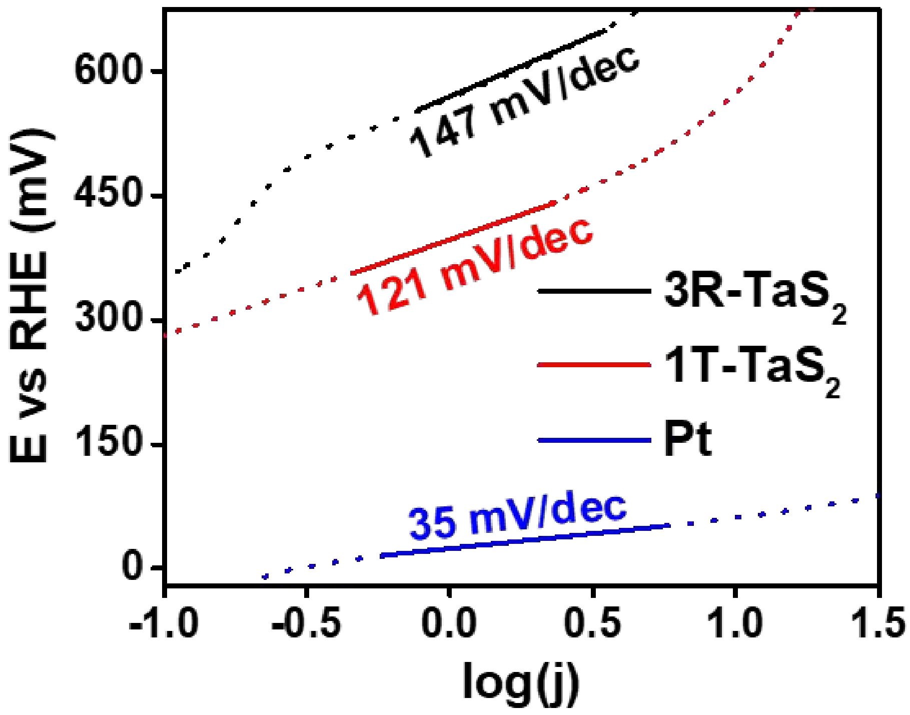

Figure S10 Tafel slopes, estimated from LSV measurements of as synthesized 3R-TaS_2 and 1T-TaS_2 flakes.

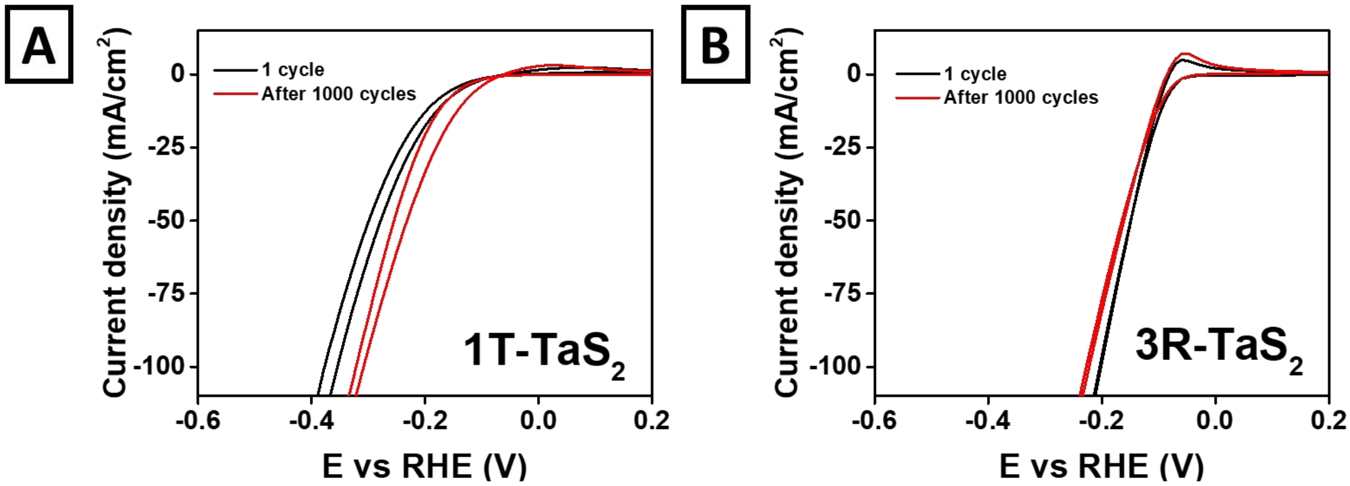

Figure S11 Cyclic stability test, performed in CVA regime (0.22 to -0.58 V vs RHE potential range) for previously activated 1T-TaS2 and 3R-TaS2 phases.

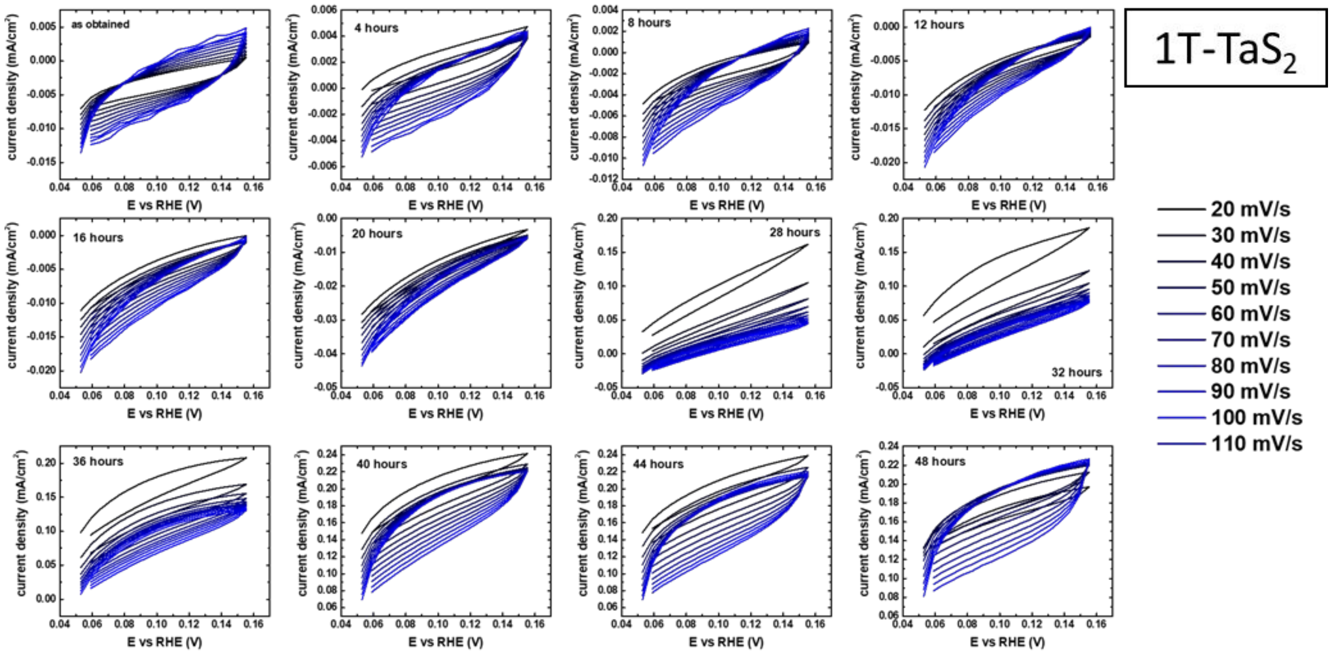

Figure S12 CVA scans, measured with different scan rates on 1T TaS_2 flakes after their activation in potentiostatic regime for different times.

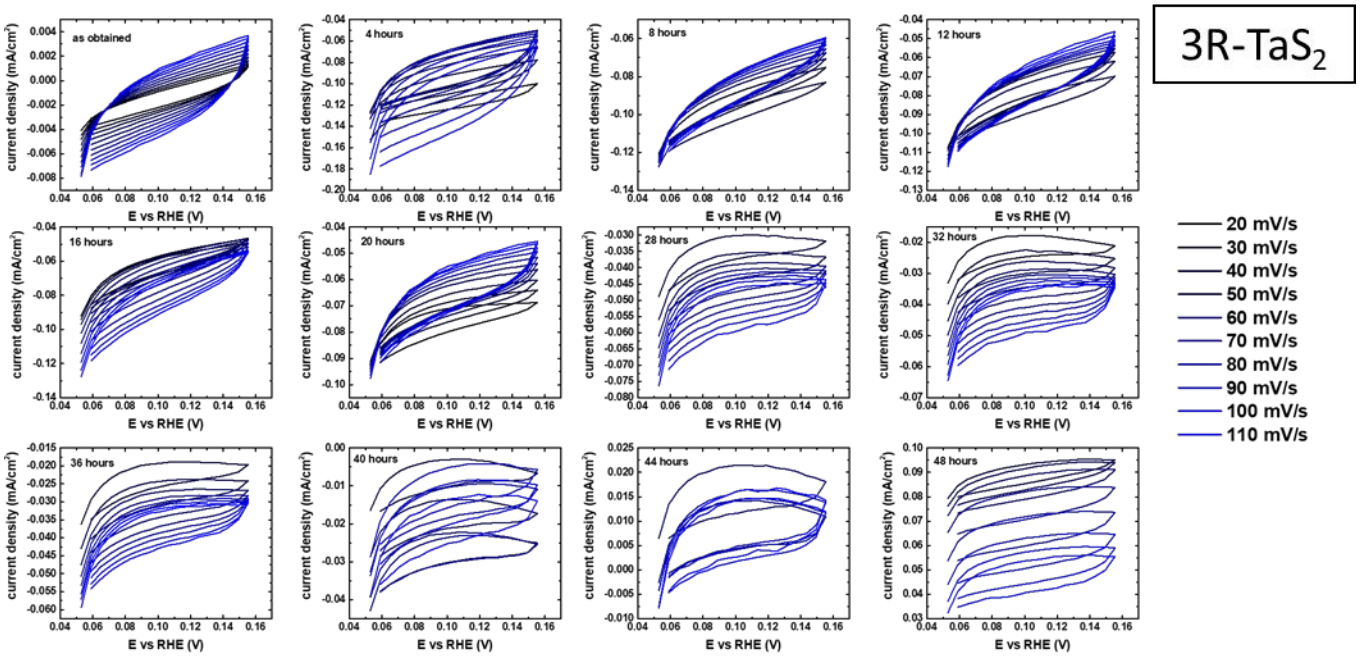

Figure S13 CV scans, measured with different scan rates on 1T TaS2 flakes after their activation in potentiostatic mode for different times.

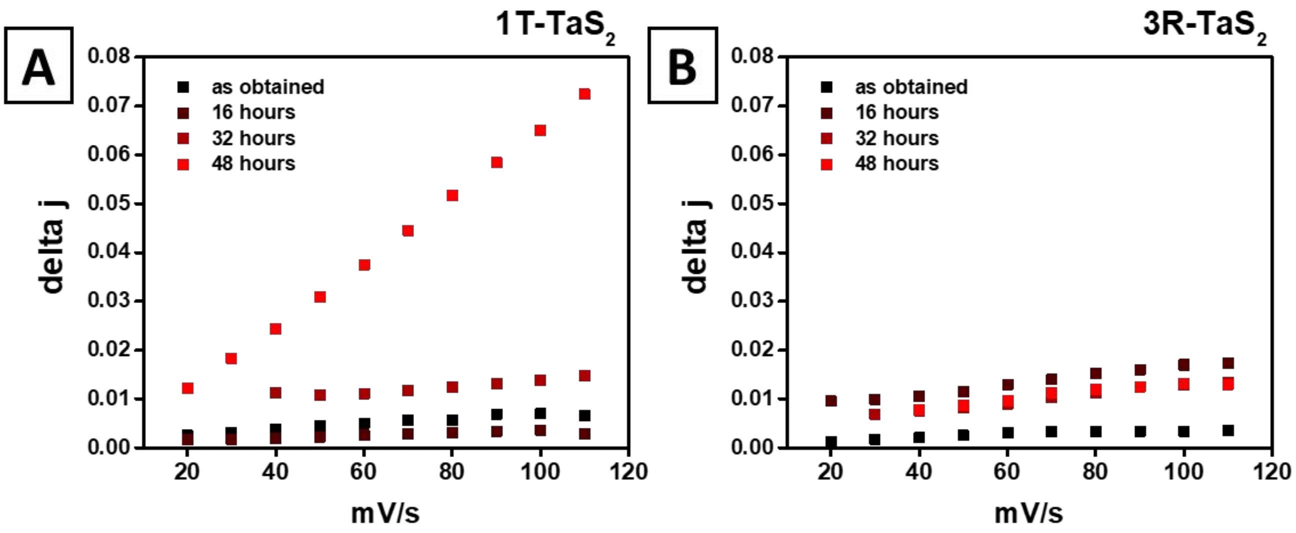

Figure S14 Difference (Δ j) in the cathodic and anodic currents plotted versus scan rate obtained after different time of samples activation for 1T-TaS_2 (A) and 3R-TaS_2 (B). The differences were calculated from CVA scan.

Figure S12, S13, and S14 – related discussion. CV measurements were performed after every 4 hours of activation, in the range 0.05 – 0.15 V vs RHE and with scan rates varying between 20 to 110 mV/s with the step of 10 mV/s (Figures S12, S13). From the obtained data, difference between current of charging and discharging (Δ j) at the half-distance was calculated and plotted versus the scan rate (see Figure S14). Inclination of the obtained slope corresponds to the capacitance of the surface, representing ECSA.

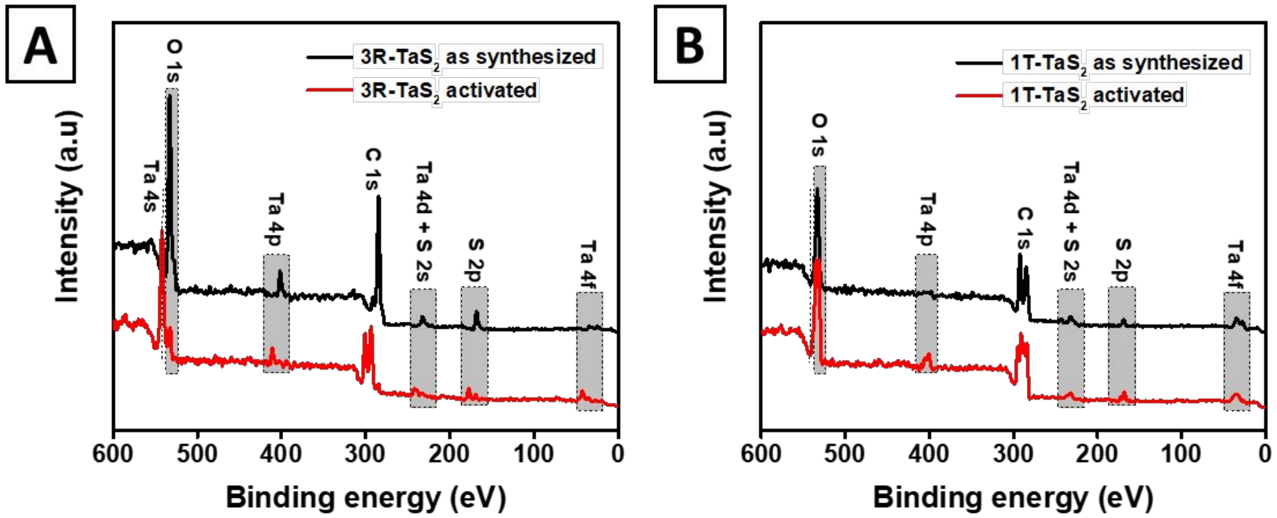

Figure S15 Survey XPS spectra (after activation) for both phases of TaS_2.

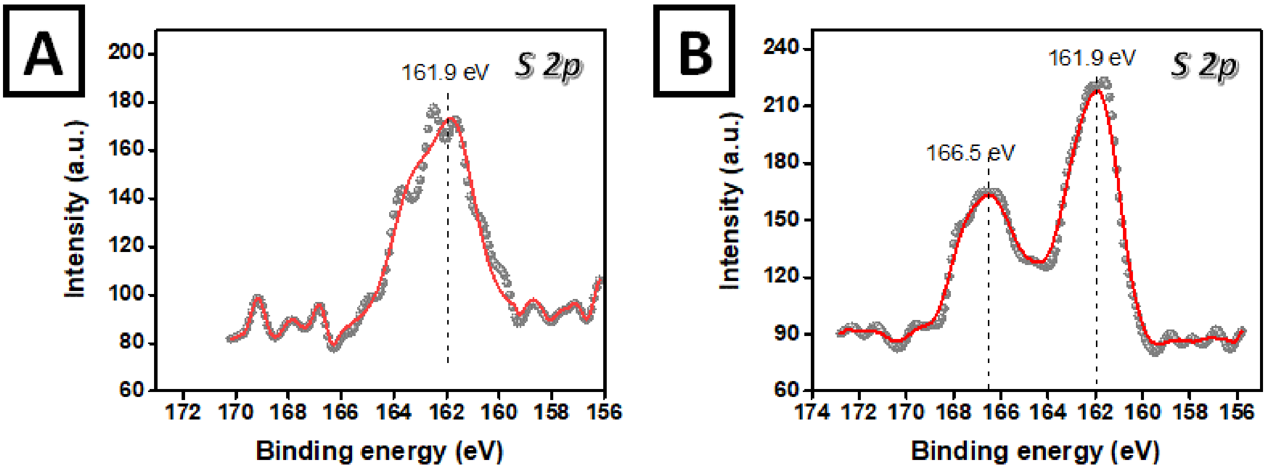

Figure S16 High resolution S(2p) spectra before and after the activation of 1T-TaS_2 phase.

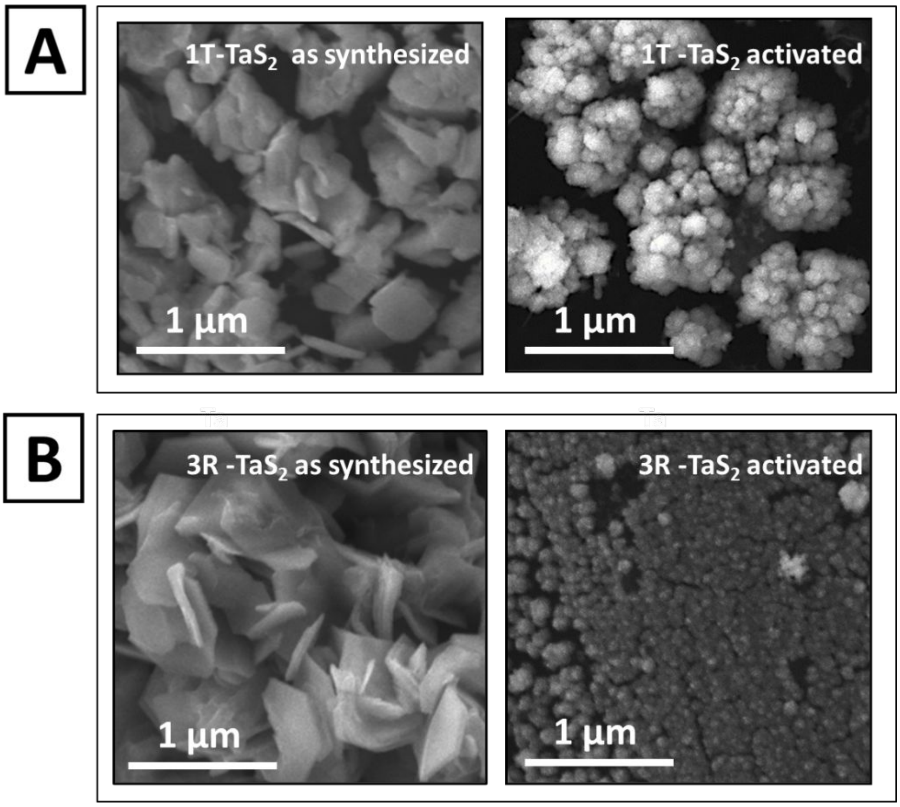

Figure S17 Comparison of surface morphology of as-synthesized (left) and activated (right) 1T-TaS_2 (A) and 3R- TaS_2 (B) flakes.

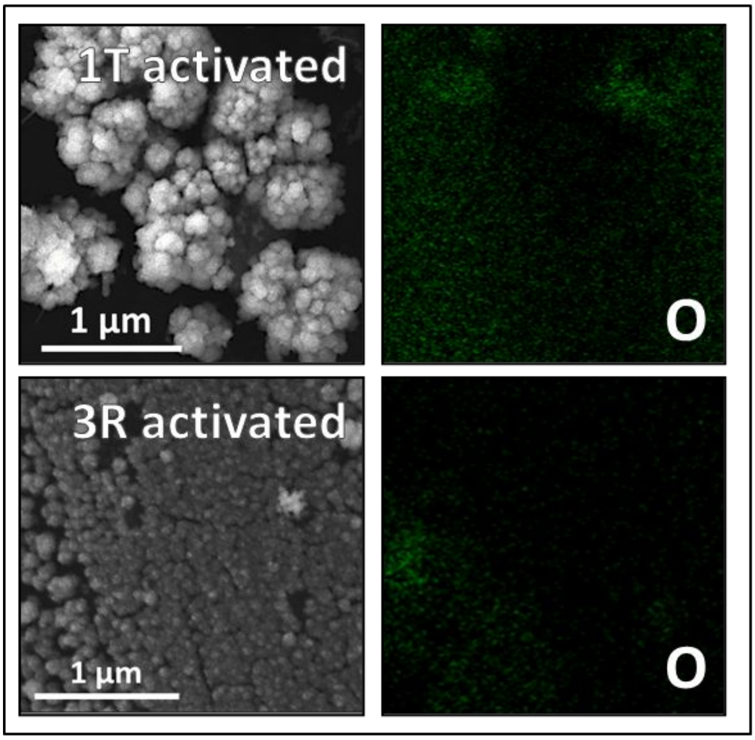

Figure S18 Additional EDX mapping (oxygen content) of the activated 3R-TaS_2 and 1T-TaS_2 flakes.

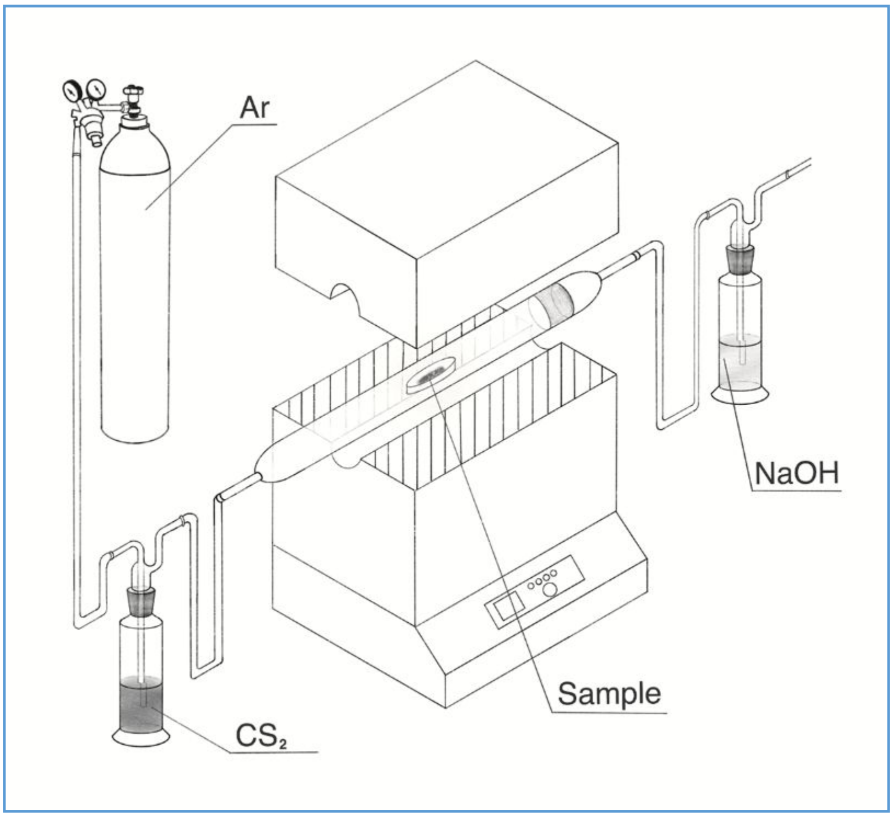

Figure S19 Schematics of the synthesis setup.

Figure S19 – related discussion. The growth of TaS_2 was performed in a setup shown in Figure S19, where a single-zone furnace (Nabertherm) equipped with a quartz tube (120 cm length and 15 cm in diameter) was used. To deliver sulfurizing agent a gas washing bottle filled with 30 mL of CS_2 was purged by Ar and connected to the quartz tube. Flow of the Ar gas was controlled by flow meter (Omega FLDA3428ST) and set to 100 sccm. The other side of the quartz tube was connected to additional gas washing bottle filled with NaOH solution to trap exhaust gases. Ta_2O_5 was placed into the quartz boat (Sample) and loaded to the quartz tube to be in the center of the furnace. Heating rate was pre-set to 10 °C/min. Once the temperature of the sulfurization was achieved it was held at constant temperature for a pre-set amount of time - 180 minutes if not specified otherwise.

References.

(S1) Wang, S.; Yang, X.; Hou, L.; Cui, X.; Zheng, X.; Zheng, J. Organic Covalent Modification to Improve Thermoelectric Properties of TaS_2. *Nat. Commun.* **2022**, *13* (1), 4401. https://doi.org/10.1038/s41467-022-32058-w.

(S2) Yu, Q.; Zhang, Z.; Qiu, S.; Luo, Y.; Liu, Z.; Yang, F.; Liu, H.; Ge, S.; Zou, X.; Ding, B.; Ren, W.; Cheng, H.-M.; Sun, C.; Liu, B. A Ta-TaS_2 Monolith Catalyst with Robust and Metallic Interface for Superior Hydrogen Evolution. *Nat. Commun.* **2021**, *12* (1), 6051. https://doi.org/10.1038/s41467-021-26315-7.

(S3) Kovalska, E.; Roy, P. K.; Antonatos, N.; Mazanek, V.; Vesely, M.; Wu, B.; Sofer, Z. Photocatalytic Activity of Twist-Angle Stacked 2D TaS_2. *npj 2D Mater. Appl.* **2021**, *5* (1), 68. https://doi.org/10.1038/s41699-021-00247-8.

(S4) Dong, Q.; Li, Q.; Li, S.; Shi, X.; Niu, S.; Liu, S.; Liu, R.; Liu, B.; Luo, X.; Si, J.; Lu, W.; Hao, N.; Sun, Y.; Liu, B. Structural Phase Transition and Superconductivity Hierarchy in 1T-TaS_2 under Pressure up to 100 GPa. *npj Quantum Mater.* **2021**, *6* (1), 20. https://doi.org/10.1038/s41535-021-00320-x.

(S5) Beydaghi, H.; Najafi, L.; Bellani, S.; Bagheri, A.; Martín-García, B.; Salarizadeh, P.; Hooshyari, K.; Naderizadeh, S.; Serri, M.; Pasquale, L.; Wu, B.; Oropesa-Nuñez, R.; Sofer, Z.; Pellegrini, V.; Bonaccorso, F. Functionalized Metallic Transition Metal Dichalcogenide (TaS_2) for Nanocomposite Membranes in Direct Methanol Fuel Cells. *J. Mater. Chem. A* **2021**, *9* (10), 6368–6381. https://doi.org/10.1039/D0TA11137F.

(S6) Wu, X.; Cai, Y.; Bian, J.; Su, G.; Luo, C.; Yang, Y.; Zhang, G. Strain Engineering and Lattice Vibration Manipulation of Atomically Thin TaS_2 Films. *RSC Adv.* **2020**, *10* (28), 16718–16726. https://doi.org/10.1039/D0RA02499F.

(S7) Najafi, L.; Bellani, S.; Oropesa-Nuñez, R.; Martín-García, B.; Prato, M.; Pasquale, L.; Panda, J.-K.; Marvan, P.; Sofer, Z.; Bonaccorso, F. TaS_2, $TaSe_2$, and Their Heterogeneous Films as Catalysts for the Hydrogen Evolution Reaction. *ACS Catal.* **2020**, *10* (5), 3313–3325. https://doi.org/10.1021/acscatal.9b03184.
(S8) Chen, H.; Si, J.; Lyu, S.; Zhang, T.; Li, Z.; Lei, C.; Lei, L.; Yuan, C.; Yang, B.; Gao, L.; Hou, Y. Highly Effective Electrochemical Exfoliation of Ultrathin Tantalum Disulfide Nanosheets for Energy-Efficient Hydrogen Evolution Electrocatalysis. *ACS Appl. Mater. Interfaces* **2020**, 8.
(S9) Zhang, M.; He, Y.; Yan, D.; Xu, H.; Wang, A.; Chen, Z.; Wang, S.; Luo, H.; Yan, K. Multifunctional 2H-TaS_2 Nanoflakes for Efficient Supercapacitors and Electrocatalytic Evolution of Hydrogen and Oxygen. *Nanoscale* **2019**, *11* (46), 22255–22260. https://doi.org/10.1039/C9NR07564J.
(S10) Zhang, J.; Wu, J.; Zou, X.; Hackenberg, K.; Zhou, W.; Chen, W.; Yuan, J.; Keyshar, K.; Gupta, G.; Mohite, A.; Ajayan, P. M.; Lou, J. Discovering Superior Basal Plane Active Two-Dimensional Catalysts for Hydrogen Evolution. *Mater. Today* **2019**, *25*, 28–34. https://doi.org/10.1016/j.mattod.2019.02.014.
(S11) Yu, Q.; Luo, Y.; Qiu, S.; Li, Q.; Cai, Z.; Zhang, Z.; Liu, J.; Sun, C.; Liu, B. Tuning the Hydrogen Evolution Performance of Metallic 2D Tantalum Disulfide by Interfacial Engineering. *ACS Nano* **2019**, *13* (10), 11874–11881. https://doi.org/10.1021/acsnano.9b05933.
(S12) Huan, Y.; Shi, J.; Zou, X.; Gong, Y.; Zhang, Z.; Li, M.; Zhao, L.; Xu, R.; Jiang, S.; Zhou, X.; Hong, M.; Xie, C.; Li, H.; Lang, X.; Zhang, Q.; Gu, L.; Yan, X.; Zhang, Y. Vertical 1T-TaS2 Synthesis on Nanoporous Gold for High-Performance Electrocatalytic Applications. *Adv. Mater.* **2018**, *30* (15), 1705916. https://doi.org/10.1002/adma.201705916.
(S13) Hu, Y.; Hao, Q.; Zhu, B.; Li, B.; Gao, Z.; Wang, Y.; Tang, K. Toward Exploring the Structure of Monolayer to Few-Layer TaS_2 by Efficient Ultrasound-Free Exfoliation. *Nanoscale Res. Lett.* **2018**, *13* (1), 20. https://doi.org/10.1186/s11671-018-2439-z.
(S14) Feng, Y.; Gong, S.; Du, E.; Chen, X.; Qi, R.; Yu, K.; Zhu, Z. 3R TaS_2 Surpasses the Corresponding 1T and 2H Phases for the Hydrogen Evolution Reaction. *J. Phys. Chem. C* **2018**, *122* (4), 2382–2390. https://doi.org/10.1021/acs.jpcc.7b10833.
